# Update for Advance CAR-T Therapy in Solid Tumors, Clinical Application in Peritoneal Carcinomatosis From Colorectal Cancer and Future Prospects

**DOI:** 10.3389/fimmu.2022.841425

**Published:** 2022-03-25

**Authors:** Siyuan Qian, Pedro Villarejo-Campos, Ismael Guijo, Sergio Hernández-Villafranca, Damián García-Olmo, Sara González-Soares, Héctor Guadalajara, Santos Jiménez-Galanes, Cheng Qian

**Affiliations:** ^1^ Department of Surgery, Fundación Jimenez Diaz University Hospital, Madrid, Spain; ^2^ Department of Surgery, Universidad Autónoma de Madrid, Madrid, Spain; ^3^ Department of Surgery, University Hospital Infanta Elena, Madrid, Spain; ^4^ Chongqing Precision Biotechnology Co. Ltd, Chongqing, China

**Keywords:** CAR (chimeric antigen receptor) T cells, solid tumor, peritoneal carciomatosis, colorectal (colon) cancer, immunotherapy

## Abstract

Latest advances in the field of cancer immunotherapy have developed the (Chimeric Antigen Receptor) CAR-T cell therapy. This therapy was first used in hematological malignancies which obtained promising results; therefore, the use of CAR-T cells has become a popular approach for treating non-solid tumors. CAR-T cells consist of T-lymphocytes that are engineered to express an artificial receptor against any surface antigen of our choice giving us the capacity of offering precise and personalized treatment. This leaded to the development of CAR-T cells for treating solid tumors with the hope of obtaining the same result; however, their use in solid tumor and their efficacy have not achieved the expected results. The reason of these results is because solid tumors have some peculiarities that are not present in hematological malignancies. In this review we explain how CAR-T cells are made, their mechanism of action, adverse effect and how solid tumors can evade their action, and also we summarize their use in colorectal cancer and peritoneal carcinomatosis.

## Introduction

Conventional treatment with chemotherapy, radiotherapy or surgery is the therapy of choice most cancer patients (1). However, conventional treatment is not sufficient in many cases of cancer, thus the use of immunotherapy has acquired an important role for treatment of relapse or refractory tumors and its use has increased, being currently a common approach in cancer treatment (2). In the same way, investigations with new immunotherapy treatments are being developed. Particularly the use of chimeric antigen receptor (CAR)-T cell has become a popular approach and, in the last decade, many studies about CAR-T cell efficacy have been published. Their results, especially in hematological malignancies, have been more than surprising, achieving complete remission in refractory or relapsed disease (3). This evidence has resulted in the approval between 2017 to 2021 of four CAR-T cell therapy by the FDA (4, 5). Most of the studies about CAR-T cells have investigated the safety and efficacy of this treatment in hematological malignancies, however only few clinical trials have studied their effect in solid tumors.

The success of CAR-T cells is due to the possibility of targeting any antigen on the cell surface of our choice, generating an immune response against the tumor cell in an MHC-independent manner for subsequent tumor elimination. CAR-T cells are made by extracting the leukocytes from the patient whole blood. Then these leukocytes are genetically modified to express chimeric receptors, offering the patient a precise, individual, unique and personalized treatment.

However, to fully understand their success we must comprehend their manufacture, how these modified cells carry out their anti-tumoral effect, which adverse effects they may produce and how they can develop tumor resistance. In this review, we summarize the current CAR-T cell therapy evidence regarding to the basis of this therapy, starting from their production and structure, their mechanism of action, adverse effects and mechanism of resistance to CAR-T therapy. We also show the current challenges in CAR-T cell therapy and in particularly in solid tumors. And finally, we delve further into their evidence in colorectal cancer and discuss the future perspectives in peritoneal carcinomatosis.

## General Information About CAR-T Cells

### Definition

Chimeric antigen receptor (CAR)-T cells are lymphocytes that have been genetically modified to express chimeric receptors that enable them to target specific surface antigens in a major histocompatibility class-independent manner. This type of modified T cells was first described by Gross et al. in 1989, though only in the last decade has this technology become more widely developed, particularly for treating hematologic malignancies (6).

Immunotherapy has become a more widely used approach in cancer treatment with the application of CAR-T cells, as they give T cells the ability to express synthetic receptors against surface antigens of our choice and thereby destroy tumor cells (7). These antigens are not limited to proteins, but rather are able to bind to carbohydrates, glycolipids, and proteoglycans (8, 9). CAR-T cells have shown promising results in the treatment of hematologic malignancies and are being used mainly to treat cancers for which the primary target is CD19, i.e., B-cell lymphoma, childhood acute lymphoblastic leukemia (ALL), and adult-onset ALL. The FDA has approved the use of 4 CAR-T cell therapies that target CD19 in the last 5 years: axicabtagene ciloleucel (trade name, *Yescarta*), tisagenlecleucel (trade name, *Kymriah*), lisocabtagene maraleucel (trade name, *Breyanzi*) and brexucabtagene autoleucel (trade name, *Tecartus*) (4, 5, 10, 11).

However, the use of CAR-T cells in solid tumors has been less widely researched, and current evidence is insufficient to determine how these cells may be used in the clinic, so this type of therapy is now limited to clinical trials (12).

**Figure 1:**
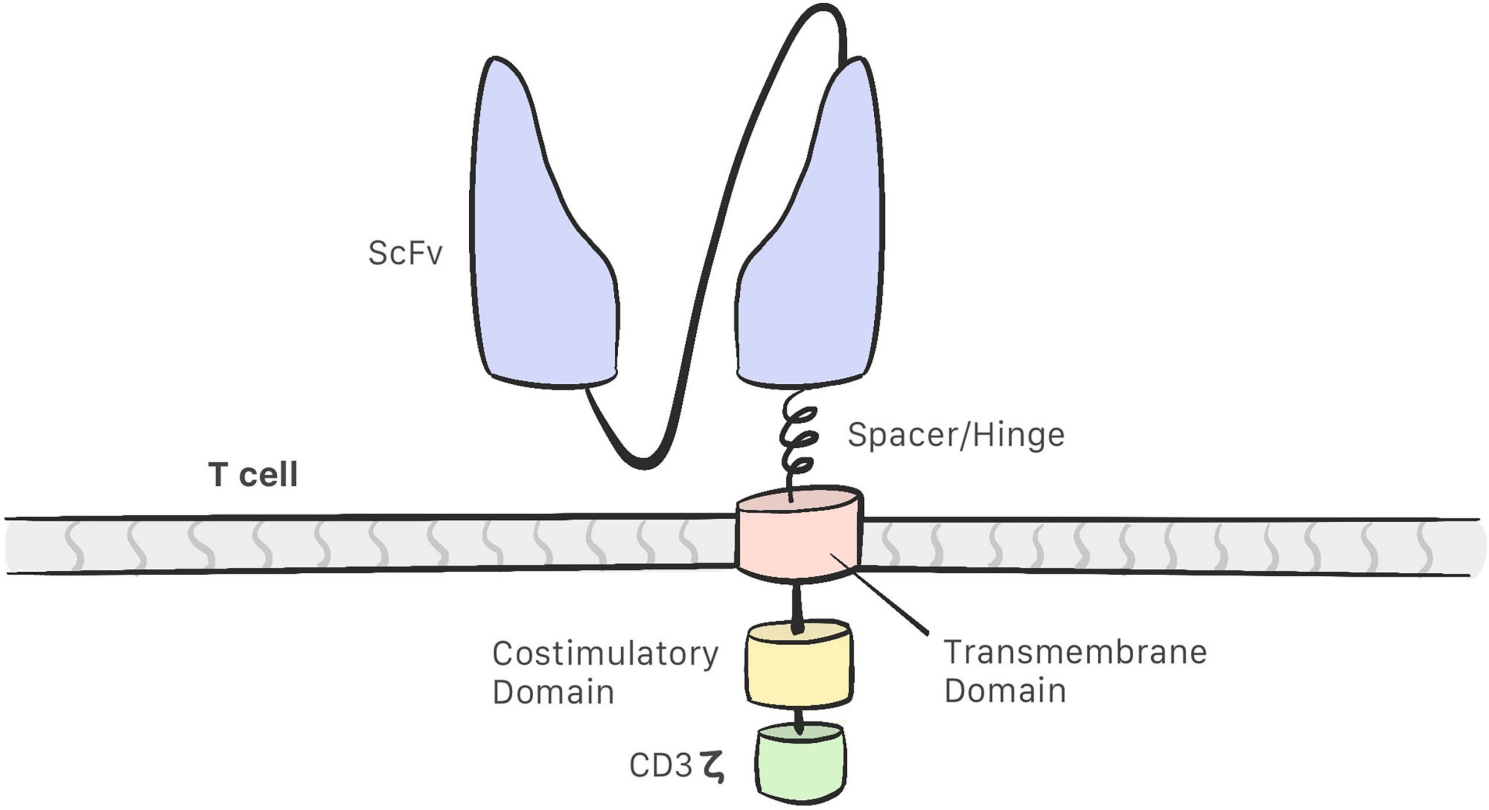
The structure of a CAR consist of a single chain variable fragment (scFv) that enables the CAR-T cell to recognise the target; a hinge/spacer that binds the scFv to the transmembrane domain and improves the scFv flexibility; a transmembrane domain that act as a link between the extracellular and intracellular portion; Coestimulatory molecules that improvesCAR-T cell efficacy and a CD3z intracelullar domain that activates the CAR-T cell.

CAR-T cells comprise the following (13–16) (**Figure 1**):

-A single chain variable fragment (scFv) tumor-targeting domain, which enables the T cell to bind to the target antigens on the cell surface.
-Hinge or spacer domain: The portion that binds the scFv to the transmembrane domain. The function of this domain is to improve scFv flexibility so that it may bind to the target. This portion is created using sequences derived from immunoglobulins. IgG1 and IgG4 are the most commonly used immunoglobulins for this purpose (9, 17).
-A transmembrane domain (TD): CD3 ζ, CD28, and CD8α have been used as membrane domains in CAR constructs. They are the link between the extracellular and intracellular portion. They also play a role in CAR-T cell efficacy and stability (17).
-Costimulatory molecules that improve CAR-T cell proliferation and persistence.
-CD3 ζ: An intracellular signaling domain that activates T cells after binding to the antigen.

**Figure 2:**
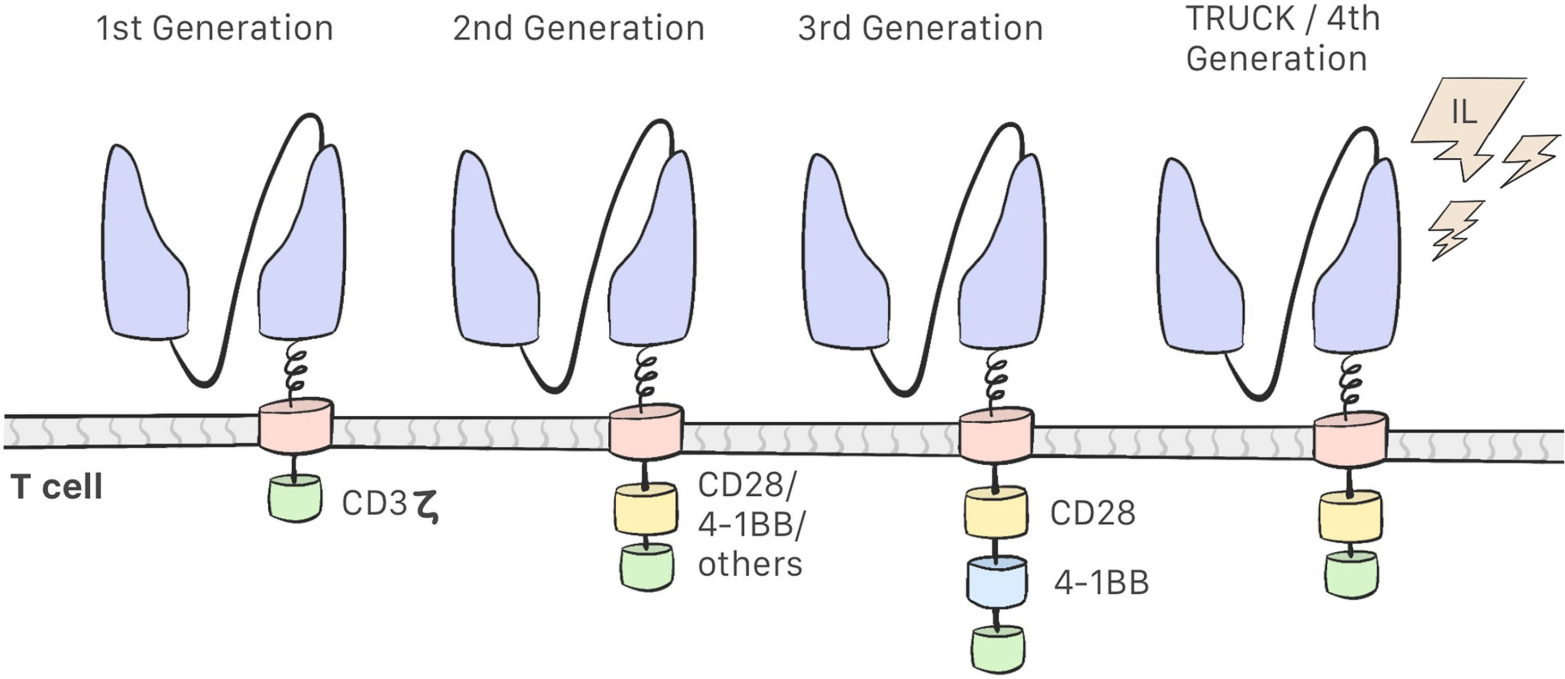
First Generation CAR-T cells consist of a scFv region and the CD3ζ intracellular domain. Second-generation CAR-T cells include a costimulatory molecules in the intracellular domain. Third-generation contains two costimulatory molecules. Fourth-generation CAR-T cell or TRUCKs secretes proinflamatory cytokines.

Development of CAR-T cells began in the late 1980s with research into these modified lymphocytes. Eshhar et al. observed that combining a variable fragment of an antibody with the constant region of the T-cell receptor endows the T-cell of the specificity of an antibody and the effector function of a cytotoxic T-cell; this resulted in the first generation of CAR-T cells, which consisted of the scFv region and the CD3ζ intracellular domain only. These cells were found to be unsuccessful in clinical trials: they were able to activate but did not proliferate, which indicated low efficacy (18–21). Second-generation CAR-T cells include costimulatory molecules in the intracellular domain, and the third generation contains 2 such molecules in the intracellular domain (12, 22) (**Figure 2**). This costimulatory molecule can be CD28, CD134 (OX40), CD137 (4-1BB), or CD27, which improve the efficacy and enhance the action, proliferation, and persistence of CAR-T cells (21–26). The fourth generation of CAR-T cells is currently being designed and have an inhibitory effect on the tumor microenvironment. These are the so-called TRUCKs (T cells redirected for universal cytokine-mediated killing), which are designed to secrete proinflammatory cytokines and recruit other immune cells, thereby improving their antitumor activity in an immunosuppressive microenvironment (12, 21, 22).

### Production

**Figure 3:**
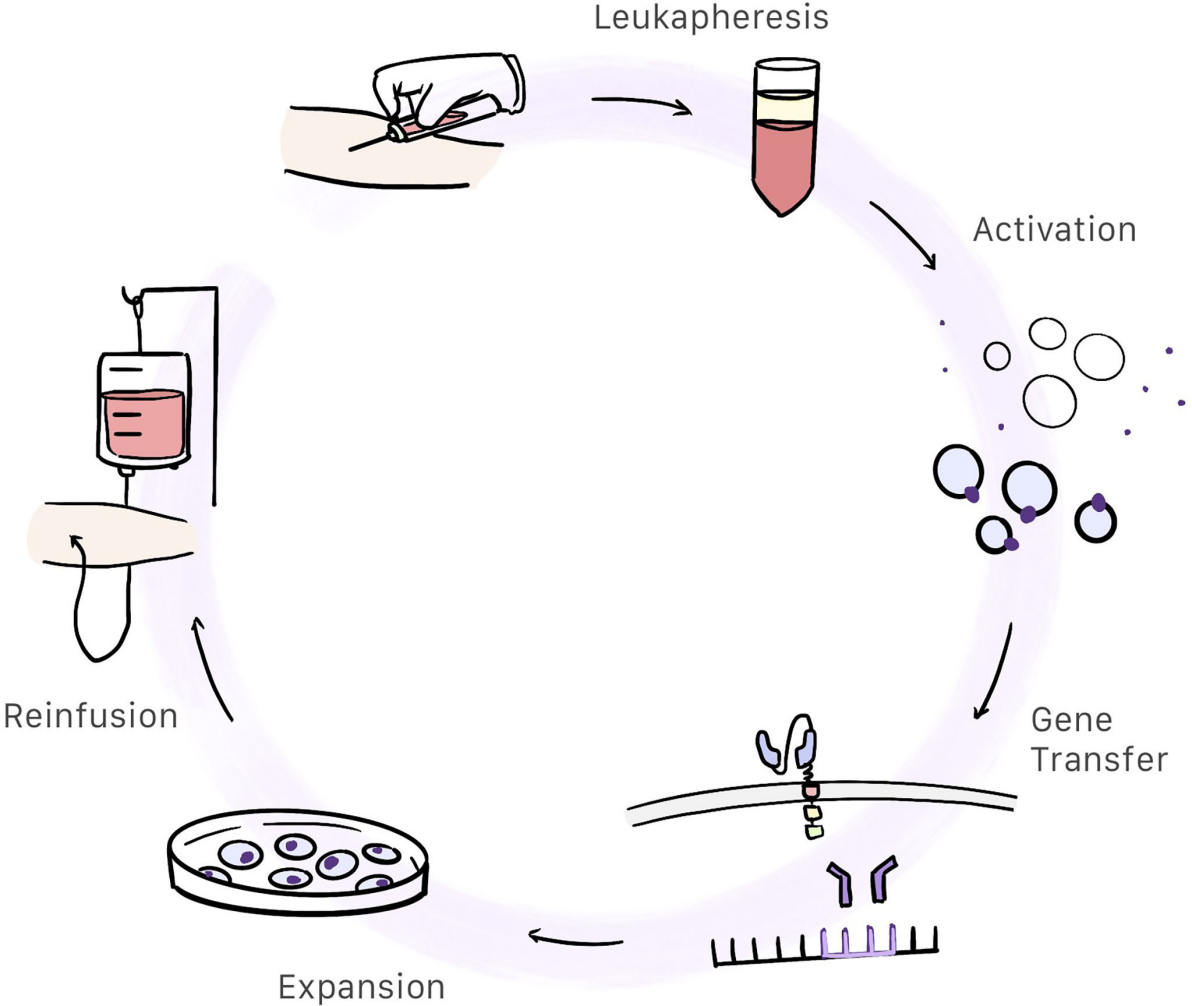
Patient whole blood is extracted and centrifugated in order to obtain the white blood cells. Then T-cells are selected and activated. Afterwards, gene transfer containing the CAR information is inserted into the activated T-cells genome resulting in the creation of CAR-T cells. These cells are expanded and after a quality control, are reinfunsed into the patient.

CAR-T cell therapy begins by obtaining a sample of whole blood from the patient. This blood will be used to extract and modify T cells so that we may produce CAR-T cells. This is a complex, work-intensive process comprising several steps (**Figure 3**). The total cost of the process is estimated at 300 000 USD.

#### Leukapheresis and Cell Washing

Patients whole blood is centrifugated and white blood cells are extracted. Anticoagulants are added throughout the process to prevent clotting (27).

#### Activation

Under physiological conditions, T cells are activated by antigen-presenting cells (APCs) and depends on the interaction of the T-cell receptor (TCR) and the major histocompatibility complex. This means of activation is a complex process requiring participation by dendritic cells, meaning that CAR-T cells are activated differently. In CAR-T cells, the primary method used to activate T cells is through anti-CD3/CD28 coated magnetic beads which are used as artificial antigen presenting particles. After T cell activation, they are removed with a strong electromagnet (27, 28). Monoclonal antibodies or artificial antigen-presenting cells can be also used to activate T cell (27).

#### Gene Transfer

When the T cell is activated, the gene containing the information for producing the chimeric antigen receptor has to be inserted in the T cell genome. Retroviral transduction, particularly with the use of lentiviral vectors, is the most commonly used method of gene transfer used in CAR-T cells (28, 29). Transposon/transposase system in plasmids can be also used as a non-viral method (30).

#### Expansion

These cells, which already express CAR, expand in a culture medium containing interleukins. This process takes between 10 days and 3 weeks to complete (9, 28).

#### Quality Control

Once the production process has completed, CAR-T cells undergo quality control, mostly to evaluate sterility (using cultures and Gram staining), purity (confirmed by the absence of replication competent viruses by means of quantitative PCR, and the absence of other type cells or endotoxins), and effectivity measured *in vitro*. Also, in this stage, the quantity of CAR-T cells produced is measured (27, 31).

### Mechanism of Action

When the scFv has been bound to the antigen, cell-destruction mechanisms are set off within the cell through the intracellular domain. T cells use 2 main pathways to mediate cytolytic effector function (14):

#### Fast-Acting. Exocytosis of Cytotoxic Granules Containing Perforin and Granzymes

This is the primary mechanism of target cell death (32). Activated T cells release cytotoxic granules containing perforins, which trigger the formation of pores in the membrane of the target cell, which will facilitate the entry of pro-apoptic granzymes inducing cell death (33). Separation of the CAR-T cell from the target cell depends specifically on cell destruction, meaning that in the absence of cell death, CAR-T cells may cause cytokine hypersecretion, leading to adverse effects such as cytokine-release syndrome (14).

#### Slow-Acting. Expression of Membrane Bound Tumor Necrosis Factor (TNF) Family Ligands

The Fas ligand (Fas-L) belongs to the TNF family. Antigen recognition by the T cell will induce Fas-L expression on the surface. Ligation between Fas and Fas-L activates a caspase cascade that initiates an induced apoptosis in the target cell (14, 34). This mechanism also has an effect on tumor cells that do not express the target antigen, though it does require previous CAR-T cell activation (35).

Independently of the direct action of T cells on tumor cells, activated CAR-T cells produce cytokines that will stimulate antitumor activity in neutrophils, macrophages, and natural killer cells. These cytokine-activated cells will trigger lysis of cells that are invisible to CAR-T cells thanks to the activation of innate immunity (9, 14). Furthermore, destruction of tumor cells will liberate tumor antigens within an immunostimulatory microenvironment, which will allow for cross-presentation by dendritic cells and generation of endogenous CD8 responses against tumor antigens which were not originally targeted (36).

### Adverse Effects Associated With CAR-T Cell Therapy

As with any treatment, use of CAR-T cells is not entirely free of adverse reactions, some of which may be life-threatening. The incidence of this toxicity varies considerably between published studies based on the types of CAR-T cells and costimulatory elements used (37).

#### Cytokine-Release Syndrome *(CRS)*

Cytokine-Release Syndrome is the main adverse effect in patients who receive CAR-T cell therapy and can result in lethal outcomes. CRS occurs in between 50% and 90% who undergoes anti-CD19 CAR-T cell therapy, mostly in the first week following the infusion (37). This effect is related to treatment response and tumor burden more so than the dose administered (21, 38). In fact, an absence of this adverse effect raises doubts as to the efficacy of the treatment and the expansion and elimination of tumor cells (12, 39). This syndrome is consequence of an over-activated immune response caused by stimulated CAR-T cells which stimulates other cells of the immune system (39, 40).

Signs and symptoms of CRS can vary widely. The most frequent symptoms are general malaise and nausea, though fever is the first symptom to present. Nonetheless, the disease may progress clinically to acute respiratory distress, acute renal failure, kidney failure, disseminated intravascular coagulation, cardiomyopathy, or even arrhythmia (12, 40). On laboratory analysis, this syndrome manifests as an increase of ferritin in serum and C-reactive protein and elevated cytokines (39, 41).

Mild cases of CRS are treated with acetaminophen and fluid therapy. Treatment with the IL-6 antagonist, tocilizumab, is indicated in patients presenting hemodynamic instability refractory to intensive fluid management and inotropic drug treatment (42). Corticosteroids may also be considered.

#### CAR T-Cell-Related Encephalopathy

Neurotoxicity can appear in 30% to 90% of patients who receive CAR-T cell therapy against CD19. Patients may suffer from headache, mild confusion or, in severe cases, cerebral edema (37). Patients with encephalopathy may exhibit disorientation, focal neurological deficit (dysphasia or motor deficits), and seizures (40, 43, 44). Encephalopathy appears between 5 and 7 days following infusion (37). Its cause is somewhat unknown, though it may be related to high levels of cytokines released, to direct neurological damage or to an off-tumor response caused by the expression of CD19 in mural cells of the brain (12, 45). A possible explanation is that CRS and the systemic inflammation may alter the permeability of the blood brain barrier allowing the arrival of cytokines and immune cells including CAR-T cells. Presence of CAR-T cells has been demonstrated in the cerebrospinal fluid of some patients (40). Neurotoxicity has only been reported in CAR-T cell therapy targeting CD19 and CD20 and until now, there is no evidence of neurotoxicity in solid tumors.

#### On-Target, Off-Tumor Response

CAR-T cells can be activated by heathy tissues that express the target antigen resulting in the destruction of non-tumoral cells. This response is more frequent in solid tumors. The intensity and frequency of this response varies according to the target and route of administration used (9, 46). For example, CD19 is widely expressed in B cells (47). The use of CD19-redirected CAR-T cells destroy not only CD19-positive neoplastic cells, but also health B cells, thus causing B-cell aplasia (48). This response can be avoided by selecting a highly specific tumor antigen but also, CAR-T cells must have high affinity and specificity for the tumor antigen chosen and an appropriate dose of CAR-T cells must be administered (9, 22).

### Barriers

#### Obstacles Related to Production

Producing CAR-T cells requires a supply of T cells. This first hurdle in the process is related to the raw material and concerns both the quality and quantity of the cells harvested. Unfortunately, CAR-T cells are currently approved only for hematologic malignancies, and some patients lack a sufficient amount of T cells from this source to create CAR-T cells. Moreover, many patients receive previous chemotherapy, which has been associated with lower quantity and quality of these T cells (49).

The remaining obstacles concern the design of CAR-T cells, particularly regarding variations in their structure, production and the time required to produce these cells (50). CAR-T cell exhaustion refers to a dysfunctional state in which the T cells lose their effector function and in which there is an increase in the number of inhibitory receptors induced by chronic stimulation such as that observed in cancer. This exhaustion may lead to treatment failure (51).

The main limitation of CAR-T cell therapy involves limited access to treatment despite the fact that this therapy has been approved by the FDA. Treatment must be carried out in specialist facilities capable of adhering to product manufacturing and administration protocols that can absorb the high cost of this therapy (50).

*The toxicity-related factors* involved in this treatment are the result of the adverse effects commented on previously in this review.

#### Tumor-Related Factors

-Antigen-negative tumor relapse: The presence of antigen-negative tumor cells from the beginning leads to the selection of cells that will not be destroyed. To develop resistance to CAR-T cells, complete antigen loss is not necessary; rather, tumor cells may become resistant with diminished numbers of antigens (50). Tumor escape may also occur due to alternative splicing events, in which the antigen continues to be present, although in an isoform that CAR-T cells are unable to recognize (52).
-Antigen-positive tumor relapse: Antigen-positive relapse may be due to defects in CAR-T cells or may result from tumor-dependent factors. The antitumor effect of CAR-T cells does not only depend on antigen recognition. Rather, it may depend on the activation of cell-destruction mechanisms. The mechanism that causes antigen-positive tumor resistance is primarily based on changes in survival and apoptosis of the tumor cell (21). A signal that induces tumor apoptosis is the TNF–related apoptosis-inducing ligand (TRAIL). The absence of TRAIL in tumor cells implies antigen-positive tumor resistance (53). Another cause of relapse concerns CAR-T cell destruction, as tumor cells may express FAS-L, which induces CAR-T cell apoptosis (54). Recurring antigen-positive tumors may respond to a second treatment with CAR-T cells. However, re-infusion of CD19-redirected CAR-T cells has not resulted in a clinical response in a number of published studies (55–57).

#### Factors Related to the Tumor Microenvironment

This factor affects particularly to solid tumors and will be explained more deeply afterward. Tumor microenvironment confers to the tumor a physical barrier composed by the tumor stroma and the extracellular matrix, a metabolic barrier and an immunological barrier contributing to an immunosuppressive state.

## Treatment of Solid Tumors with CAR-T Cells

In light of the surprising results obtained in CAR-T cell therapy in hematologic malignancies, it comes as no surprise that research on their use in solid tumors is now under way. Multiple targets in solid tumors are currently being investigated for CAR-T cell therapy, including human epidermal growth factor receptor 2 (HER-2) for breast, ovarian, lungs, and pancreas pancreas; carcinoembryonic antigen (CEA) for digestive tumors and tumors of the lung; disialoganglioside 2 (GD2) for neuroblastoma; IL-13Rα for glioblastoma; epidermal growth factor receptor (EGFR) for pancreatic cancer and glioblastoma (58); MUC for pancreatic, gastric, and ovarian cancer; mesothelin for mesothelioma; and prostate-specific membrane antigen (PMSA) for prostate cancer. However, current evidence is lacking to transfer this approach to the routine clinical practice, and the results obtained to date are less promising when compared to applications in hematologic malignancies.

Solid tumors respond differently owing to the presence of barriers that do not affect liquid tumors. Directing CAR-T cells against solid tumors requires presence of a specific antigen on the tumor surface. Once administered, CAR-T cells must migrate toward the tumor and infiltrate it. Subsequently, CAR-T cells must reach the tumor cell and recognize it in within a hostile microenvironment characterized by oxidative stress, acidic pH, hypoxia, nutritional depletion, presence of inhibitory factors and cytokines and also immunosuppressive cells (59). Regarding to the adverse effects of CAR-T cell therapy in solid tumors, they are the same as in hematologic malignancies aforementioned taking into account that on-target, off-tumor response varies depending on the target and if it is administered regionally, local reaction may appear. Therefore, current challenges in CAR-T cell therapy in solid tumors focus on target selection, CAR-T cells migration and tumor microenvironment.

### Current Challenges in Solid Tumors

#### Target-Antigen Selection

To reduce on-target, off-tumor toxicity, the target antigen must be present on the surface of all tumor cells and be absent from healthy cells. Locating a specific tumor-associated antigen (TAA) poses a challenge in solid tumors. Surface oncofetal antigens are a good target for CAR-T cell therapy, as their expression is limited to tumor cells (7).

Additionally, TAAs on solid tumors are more heterogeneous than those of hematologic malignancies (60, 61). Use of CAR-T cells for treating hematologic malignancies derived from B cells have the advantage that practically all the B-cell line (whether they are tumor cells or not) expresses CD19, which makes this a perfect therapy target. However, CD19-redirected CAR-T cells also destroy healthy cells, thus causing B-cell aplasia, which makes the host prone to opportunistic infections (48). Therefore, finding a marker with these characteristics in solid tumors is fundamental.

#### Migration

Once CAR-T therapy has been administered, these engineered cells must penetrate the tumor and reach tumor cells. Hematologic malignancies are free of this difficulty, as both CAR-T cells as well as their target cells are hematopoietic, which makes them tend to migrate to similar locations (62). Chemokines secreted by solid tumor cells, such as CCL2, frequently do not match the chemokine receptors in CAR-T cells. Induction of expression of these receptors such as CCR2b favors migration to the tumor (63). Tumor cells also express chemokines such as CXCL5, which attracts myeloid-derived suppressor cells (MDSCs), which have an immunosuppressive effect (64). One possible solution to this is to administer CAR-T cells locally.

#### Tumor Microenvironment

The tumor microenvironment provides the tumor with a series of barriers that hinder the action of CAR-T cells.

##### Physical Barrier

Even after the CAR-T cells have migrated properly, effector cells encounter a physical barrier as the tumor stroma prevents them from infiltrating the tumor. Destroying this stroma by generating CAR-T cells that are able to secrete enzymes that degrades the stroma (65); or as an alternative, local collagenase may be administered, favoring this way CAR-T cell infiltration (66).

##### Metabolic Barrier

Glucose is the preferred energy source of tumor cells, causing an increase in lactic acid production. This causes nutritional depletion, lactate elevation (and, thus, acidic pH), reduced glucose and increased oxidative stress, thus inhibiting T-cell proliferation and cytokine production (7, 62, 67).

##### Immunologic Barrier

Multiple soluble inhibitory factors exist in the tumor microenvironment. Netwick et al. describe presence of prostaglandin E2 (PGE2), a molecule produced by tumor cells and macrophages, as well as high levels of adenosine, which are further increased during hypoxia. Both PGE2 and adenosine inhibit T-cell proliferation (7). TGF-β (transforming growth factor β) and IL-10 are secreted by tumor cells and immunosuppressive cells (62). TGF-β favors tumor progression and metastasis and has a direct negative effect on T-cell differentiation and cytotoxic function (68). IL-10 inhibits the activation of T cells (69). Blocking of TGFβ signaling or the use of TRUCKs may favor a response by CAR-T cells.

In addition to soluble inhibitory factors, the tumor microenvironment contains immunosuppressive cells such as regulatory T cells (Tregs), myeloid-derived suppressor cells (MDSCs), tumor-associated macrophages (TAMs), and tumor-associated neutrophils (TANs), which inhibit the function of effector T cells. Tregs inhibit T-cell function through cell-cell contact and soluble factors such as TGFβ and IL-10 (70). MSDCs, TAMs, and TANs inhibit the immune response by producing TGF-β and PGE2 (71).

Another inhibitory factor involves the expression of programmed death-ligand 1 (PD-L1) by tumor and immunosuppressive cells and the expression of cytotoxic T lymphocyte associated antigen-4 (CTLA-4; also known as B7-1/B7-2) by these cells; both reduce T-cell function. The PD receptor is located on the membrane of T cells. PD-L1 expression by health cells prevents them from being destroyed by T cells. Cancer cells and Tregs express PD-L1, which enables them to inhibit the action of CAR-T cells and also favors CAR-T cell apoptosis. CTLA4 is a receptor expressed by T cells, though when stimulated by B7-1/B7-2 inhibits the effector functions of T cells (72). Lastly, Fas-L expression by tumors induce T-cell apoptosis (54). Adjuvant CAR-T cell administration with monoclonal antibodies that block PD1 and CTLA4 may increase the antitumor effect of CAR-T cells (59, 73).

### Future Prospects

Many studies have shown that when used as monotherapy, CAR-T cells have limited efficacy against solid tumors. However, these results may be biased by the sample of patients who have received previous failed treatments and who then initiate CAR-T cell therapy in a poor physical state and with aggressive tumors (74).

#### Chemotherapy

Combined therapy consisting of chemotherapy and CAR-T cells has a synergistic effect. Chemotherapy reduces tumor burden and plays an immunomodulatory role when administered at low doses; this benefits the inhibition of suppressant immune cells, as they are more sensitive that cytotoxic T cells (75) and reduce autoimmunity by prolonging the persistence of CAR-T cells (74). Chemotherapy with low-dose carboplatin also sensitizes tumor cells to immunotherapy, thereby increasing the antitumor effect of CAR-T cells (76). Lastly, the cytotoxic effect of chemotherapy facilitates tumor-antigen recognition and presentation (74).

#### Radiation Therapy

Radiotherapy induces tumor necrosis and apoptosis, which favors the maturation and activation of dendritic cells and the presentation of antigens (77). Following radiation, INF-ϒs and damage-associated molecular patterns (DAMPS) are released, attracting immune effector cells, which boosts migration and infiltration of the tumor, and also increases MHC class-I molecules expression (78).

Radiation is also followed by an immune-mediated antitumor response targeting distant tumors, which affects primary-tumor metastasis; this phenomenon is referred to as the abscopal effect (79).

#### Combination With Other Types of Immunotherapies

Optimization of CAR-T cells allows for enhanced antitumor function.

The effect of CAR-T cells is enhanced when administered in conjunction with TRUCKs. TRUCKs were designed to secrete proinflammatory cytokines that increase their action in an immunosuppressive tumor microenvironment; in particular, TRUCKs that produce IL-12 improve the cytotoxicity of T cells and favor the expansion and secretion of cytokines, thus conferring resistance to Tregs (80).

Creating CAR-T cells capable of recognizing 2 antigens would prevent tumor escape. Dual CAR-T cells are T cells that express 2 CARs against different antigens and are only activated when both antigens are expressed on the tumor surface (81). Tandem CARs (TanCARs) are T cells that express a CAR that is capable of recognizing two different antigens, which are activated by either receptor (82).

It has been shown that adding anti-PD1 monoclonal antibodies or CAR-T cells capable of secreting anti-PD-L1 can block the inhibitory effect of these receptors and allow CAR-T cells to function, thus improving their efficacy and persistence (83, 84). Anti-CTLA-4 antibodies have also been shown to increase T-cell activity (85).

#### Local Treatment With CAR T Cells

Most studies using CAR-T cells in solid tumors have been carried out by means of systemic administration of these T cells. This body of research has reported limited efficacy owing to the low capacity of CAR-T cells to migrate to the tumor site. As a result, local application of CAR-T cells would likely increase tumor penetration. However, this approach is limited by its high technical complexity.

Brown et al. showed that local infusion of anti-IL13Ra2 CAR-T cells into the resection cavity of 3 patients with glioblastoma was both safe and feasible (86). Brown and her coauthors later published a case report of a single patient with recurrent multifocal glioblastoma who received multiple local infusions of CAR-T cells targeting IL13Ra2, observing that administration into the resected cavity controlled local relapse and progression of glioblastoma in distal sites; on the other hand, the authors indicated that intraventricular infusions led to regression of all tumors of the central nervous system (58).

Local CAR T-cell administration may provide a solution to the problem of CAR T-cell migration to the tumor site, thus improving penetration. Doing so would prevent adverse effects associated with on-target, off-tumor responses and lower the occurrence of CRS (87).

## Treatment of Colorectal Cancer with CAR-T Cells

Colorectal cancer (CRC) is the third most common type of tumor and the second leading cause of cancer-related death (88). As a result of screening initiatives, many patients with colorectal tumors are diagnosed at an early stage of the disease, for which curative treatment is available. However, approximately 20% of these patients present metastatic disease on diagnosis, and many cases of CRC may recur following conventional therapy (89). In these patients with distant tumors, chemotherapy can make it possible for these patients to have a mean survival of 20-30 months (90). Despite the many available treatment lines, the survival rate continues to be low. Therefore, CAR-T cells hold potential as therapy for these patients.

To date, all studies on CAR-T cell therapy in CRC patients have been performed in patients with metastatic disease, as conventional approaches to localized tumors have demonstrated good outcomes. The first such study was conducted in patients with CRC and metastasis of the liver. The investigation consisted of 2 trials using first-generation CAR-T cells targeting tumor-associated glycoprotein-72 (TAG-72), an oncofetal mucin overexpressed by most human epithelial adenocarcinomas, with expression predominantly restricted to tumor cells. In one of the trials, cells were administered intravenously in escalating-dose and in the other infusion was administered through the hepatic artery. The study concluded that CAR-T cell therapy is safe despite the migration difficulties found for these cells (91).

Carcinoembryonic antigen (CEA), another attractive target for CAR-T cell therapy, is a marker for gastrointestinal cancer that is widely expressed in CRC. This marker is not detected in most normal adult tissues and is only expressed at very low levels in the luminal epithelia of the gastrointestinal tract and lung tissue, which causes it to go undetected by immune cells, while in tumor cells this antigen loses its polarity, causing it to be expressed on the entire cell surface (92).

The first therapy to use CAR-T cells redirected against CEA employed hepatic transarterial administration to deliver second-generation cells to patients with liver metastases. The authors of the study found that this type of therapy is safe and also provided evidence of the presence of CAR-T cells in liver and tumor tissue (93).

Another clinical trial studied systemic administration of CAR-T cells directed against CEA in patients with metastatic CRC. The authors reported that the patients tolerated the treatment well, even at high dose levels (up to 10^8^ cells/kg), and that the treatment helped control the disease. The study consisted of administering second-generation CAR-T cells in 10 patients with progressive metastatic CRC. Following treatment, 7 patients had stabilized disease, 2 of whom remained stable for over 30 weeks, and another 2 patients had a reduction in the size of their tumors (38). The effect of these cells depends on the ability of the CAR-T cells to expand and persist; cell loss may lead to tumor relapse (94). The antitumor effect and expansion of CAR-T cells is determined by the presence of immunosuppressive factors; these factors can be attenuated with lymphodepletion by cyclophosphamide/fludarabine chemotherapy (38). CAR-T cells that persist in the body may be capable of eliminating tumor cells in the event of rechallenge (95). Although use of TCRs redirected against CEA has been associated with colitis, none of the patients studied developed this adverse effect, even at high doses (96).

This indicates that anti-CEA CAR-T cell therapy is safe. The adverse effects related to CRS are mild and easily manageable (38, 93, 97). Therapy consisting of CEA-specific CAR-T cells administered simultaneously alongside IL-12 has been shown to increase antitumor activity and favor CAR-T cell proliferation (98).

Meanwhile, other targets are being investigated as approaches in CRC therapy. Epithelial-cell adhesion molecule (EpCAM) CAR-T cells may have antitumor effects. EpCAM is expressed on most carcinomas and is associated with E-cadherin–mediated adhesion to support tumor dissemination (99). EGFR CAR-T cells (*epidermal growth factor receptor*) have shown antitumor activity *in vivo* (100). HER-2 is another target expressed on many tumors and in approximately 15% of CRCs (101). Animal models have shown good results (95), though in one clinical trial caused acute respiratory failure syndrome (102). A phase-I clinical trial used CD133-directed CAR-T cells in patients with hepatocellular carcinoma, pancreatic carcinoma, or CRC, observing an antitumor response (103).

Since early stages of CRC can be managed by conventional treatment, the use of CAR-T cells in CRC could only offer benefits in metastatic disease. Thus, all effort must be focused in treating distant metastasis. To date, CEA is the most promising target for treating disseminated CRC. However, multiples targets are being investigated that have showed promising results.

## Treatment of Peritoneal Carcinomatosis with CAR-T Cells

The peritoneum is a common site of dissemination in CRC, forming part of the natural evolution of the disease in up to 40% of CRC patients (104). Between 5% and 10% of peritoneal metastases are detected on diagnosis (105). This entity has a high mortality rate and a mean survival of 6 months if left untreated (106).

As aforementioned, solid tumors may encounter some obstacles that are not present in hematological malignancies. CAR-T cells migrate to similar locations as hematological malignancies due to their similar origin. This situation does not happen in solid tumors, thus CAR-T migration is suboptimal. To improve CAR-T migration to the peritoneal metastases, local treatment with intraperitoneal instillation might be a possible solution.

Katz et al. used second-generation CAR-T cells targeting CEA to treat peritoneal carcinomatosis in a murine model, demonstrating that local peritoneal infusion of CAR-T cells was superior to systemic administration. Intraperitoneal injection of CAR-T cells was associated with greater tumor reduction when compared to intravenous infusion and showed a lasting effect, protecting the host from rechallenge and from tumors in extraperitoneal sites (107).

In a similar study, another group used second-generation TAG72-CAR-T cells in an animal model of peritoneal ovarian carcinomatosis. Regional intraperitoneal delivery showed better results than systemic delivery, with increased tumor reduction and overall survival, which were higher after repeated infusion (108).

Another study of peritoneal carcinomatosis observed that repeat intraperitoneal delivery of EpCAM CAR-T cells using mRNA-mediated transfection produced an inhibitory effect on tumor growth and, given that these cells express CAR-T cells *via* mRNA, their expression is temporary, which increases their degree of safety (109). Occasionally, CAR-T cells may fail to recognize tumor targets due to an absence of the target epitope caused by gene deletions or alternative splicing; in these cases, a second infusion of CAR-T cells may not have any added effect (52, 103).

Another challenge present in the treatment of solid tumors is due to the tumor microenvironment components that offers a resistance preventing CAR-T cells to reach the tumor cells. One of these components is the tumor stroma that confers a physical barrier. Particularly in the case of peritoneal metastases, they present high levels of collagen in their extracellular matrix. Thus, the destruction of these collagen fibers using intraperitoneal collagenase followed by intraperitoneal CAR-T cells instillation may favor CAR-T cells infiltration to the tumor and enhance their activity (66).

Therefore, when compared to systemic administration, regional intraperitoneal delivery of CAR-T cells to treat peritoneal carcinomatosis produces a greater antitumor effect, added protection against rechallenge, and protection in extraperitoneal tumor sites. This effect may be amplified with repeat infusions. Peritoneal administration allows increased local concentration of effector cells, which triggers a local immune response in the peritoneal cavity in addition to minimizing the adverse systemic effects caused by CAR-T cells (109, 110). This, however, does not influence the effect of CAR-T cells on distant tumors, as it is not a result of the direct action of CAR-T cells, but rather a phenomenon resembling the abscopal effect (107). This mechanism, which is seen in radiotherapy, consists of tumor-antigen secretion by the cancer cells destroyed by CAR-T cells, allowing for cross-presentation by dendritic cells, which mount an immune response against antigens other than those targeted by the CAR-T cell (14). Furthermore, activation of CAR-T cells releases cytokines, which stimulate the innate immune response (36).

## Conclusions and Future Prospects

Despite that CAR-T cells therapies are being widely investigated and had reached excellent result in hematological malignancies; solid tumors have not achieved the expected effect and its efficacy is still unclear. This lack of efficacy is due to some important hurdles that are present in solid tumors and need to be resolved. The tumor microenvironment entails the main difficulty for CAR-T cell to carry out their function due to the physical barrier and immunosuppressive microenvironment. Numerous studies are trying to improve CAR-T cell efficacy by prolonging their persistence, trafficking, tumor infiltration and tumor elimination by means of using different costimulatory molecules, CAR-T capable of secreting proinflammatory cytokines or capable of detecting two different antigens. Also, combination therapy with other immunotherapies, chemotherapy or radiotherapy may improve their results.

Recent data show that peritoneal carcinomatosis can be treated with local instillation of CAR-T cells with promising result and less systemic adverse effects. We suggest treating peritoneal carcinomatosis with combination therapy using local instillation of collagenase for treating the tumor stroma followed by intraperitoneal CAR-T cell instillation. We believe that this approach could improve the efficacy of CAR-T cell therapy in peritoneal carcinomatosis. But also, its combination with other immunotherapy such as anti-CTLA-4 or anti-PD1 monoclonal antibodies offers a wide field of investigation.

## Author Contributions

SQ contributed in writing the original draft preparation. SQ, PV-C, IG, and CQ wrote de manuscript. SH-V, SG-S, HG, SJ-G, DG-O, and CQ reviewed the manuscript. CQ corrected the manuscript. SQ and PV-C edited the manuscript before submission. CQ supervised the manuscript. All authors contributed to the article and approved the submitted version.

## Conflict of Interest

Author CQ is employed by Chongqing Precision biotechnology Co. Ltd.

The remaining authors declare that the research was conducted in the absence of any commercial or financial relationships that could be construed as a potential conflict of interest.

## Publisher’s Note

All claims expressed in this article are solely those of the authors and do not necessarily represent those of their affiliated organizations, or those of the publisher, the editors and the reviewers. Any product that may be evaluated in this article, or claim that may be made by its manufacturer, is not guaranteed or endorsed by the publisher.
